# Interplay between Bladder Microbiota and Urinary Antimicrobial Peptides: Mechanisms for Human Urinary Tract Infection Risk and Symptom Severity

**DOI:** 10.1371/journal.pone.0114185

**Published:** 2014-12-08

**Authors:** Vanessa Nienhouse, Xiang Gao, Qunfeng Dong, David E. Nelson, Evelyn Toh, Kathleen McKinley, Paul Schreckenberger, Noriko Shibata, Cynthia S. Fok, Elizabeth R. Mueller, Linda Brubaker, Alan J. Wolfe, Katherine A. Radek

**Affiliations:** 1 The Burn and Shock Trauma Research Institute at Loyola University Chicago, Health Sciences Division, Maywood, Illinois, United States of America; 2 Department of Microbiology and Immunology at Loyola University Chicago, Health Sciences Division, Maywood, Illinois, United States of America; 3 Stritch School of Medicine at Loyola University Chicago, Health Sciences Division, Maywood, Illinois, United States of America; 4 Infectious Disease and Immunology Research Institute at Loyola University Chicago, Health Sciences Division, Maywood, Illinois, United States of America; 5 Department of Obstetrics/Gynecology, Division of Female Pelvic Medicine and Reconstructive Surgery at Loyola University Chicago, Health Sciences Division, Maywood, Illinois, United States of America; 6 Department of Pathology at Loyola University Chicago, Health Sciences Division, Maywood, Illinois, United States of America; 7 Department of Urology at Loyola University Chicago, Health Sciences Division, Maywood, Illinois, United States of America; 8 Department of Surgery at Loyola University Chicago, Health Sciences Division, Maywood, Illinois, United States of America; 9 Department of Microbiology and Immunology, Indiana University School of Medicine, Indianapolis, Indiana, United States of America; 10 Department of Biological Sciences, University of North Texas, Denton, Texas, United States of America; 11 Department of Computer Science and Engineering, University of North Texas, Denton, Texas, United States of America; University of Toledo School of Medicine, United States of America

## Abstract

Resident bacterial communities (microbiota) and host antimicrobial peptides (AMPs) are both essential components of normal host innate immune responses that limit infection and pathogen induced inflammation. However, their interdependence has not been investigated in the context of urinary tract infection (UTI) susceptibility. Here, we explored the interrelationship between the urinary microbiota and host AMP responses as mechanisms for UTI risk. Using prospectively collected day of surgery (DOS) urine specimens from female pelvic floor surgery participants, we report that the relative abundance and/or frequency of specific urinary microbiota distinguished between participants who did or did not develop a post-operative UTI. Furthermore, UTI risk significantly correlated with both specific urinary microbiota and β-defensin AMP levels. Finally, urinary AMP hydrophobicity and protease activity were greater in participants who developed UTI, and correlated positively with both UTI risk and pelvic floor symptoms. These data demonstrate an interdependency between the urinary microbiota, AMP responses and symptoms, and identify a potential mechanism for UTI risk. Assessment of bacterial microbiota and host innate immune AMP responses in parallel may identify increased risk of UTI in certain populations.

## Introduction

Urinary tract infections (UTIs) are the most common type of bacterial infection, frequently nosocomial infections, and have estimated treatment costs exceeding $1 billion/year. Some UTI risk factors include female gender, older age, and having surgery for pelvic organ prolapse (POP) and/or urinary incontinence (UI) [1]–[3].

Pelvic floor disorders, including POP and UI, are also common. Approximately one quarter of women experience a pelvic floor disorder during their lifetime, and approximately one in ten women undergo POP/UI surgery annually [4], [5]. Despite clinical care strategies designed to eradicate potential pathogens prior to POP/UI surgery, women who have undergone POP/UI surgery are more likely to develop a UTI in the early post-operative period, presumably due to urinary tract instrumentation, compared to males or females undergoing other surgical procedures [6]. Even with sterile surgical technique, appropriate urinary catheter insertion methods, quality catheter hygiene and prophylactic antibiotic regimens, a significant proportion (∼20% of urogynecologic patients) experience UTI within the first 6 weeks after POP/UI surgery and catheter removal [7]. Despite the clear increased risk of UTI for patients undergoing POP/UI surgery, clinicians lack scientifically valid methods to identify, and ultimately treat, specific patients with an increased UTI risk.

Emerging evidence challenges the current paradigm that the bladder is a sterile microenvironment, and reveals that live bacteria are present in the bladder [8]–[12], even in ‘culture-negative’ patients. We recently identified bacteria in the bladders (urinary microbiota) of continent and incontinent adult females. Using a high-throughput culture-independent 16S rDNA sequencing approach, we found compelling evidence that bacteria are often present in urine from ‘culture-negative’ women who do not have a clinical UTI [13] and used a novel expanded quantitative urine culture protocol to cultivate many bacterial species that are often missed by conventional cultivation-dependent approaches [14]. We also reported DNA-based evidence that certain urinary microbiota characteristics associate with increased urinary urgency incontinence episodes, but fewer overt post-instrumentation UTIs [15]. More recently, we determined that women with or without urgency urinary incontinence exhibit vastly different urinary microbiomes using both 16S gene sequencing and culture-based methods. We determined that the urgency urinary incontinence microbiome was composed of increased Gardnerella and decreased Lactobacillus compared to controls. Several genera were more frequently cultured from the urgency urinary incontinence cohort, as compared to the microbiome of those individuals without urgency urinary incontinence, with *Lactobacillus gasseri* identified more robustly detected in the urgency urinary incontinence cohort and *Lactobacillus crispatus* more robustly detected in controls [16]. These data raise the possibility that the urinary microbiota play a role in maintaining urinary microbial equilibrium, and that these differences likely contribute to the clinical manifestations of pelvic floor pathology, including UTI.

Given the discovery and confirmation of a female urinary microbiome, an understanding of the role of the innate immune system is necessary, as it is a biologically plausible mechanism to maintain urinary microbial equilibrium. Antimicrobial peptides (AMPs) are a structurally diverse group of peptides that are fundamental components of the innate immune system. AMPs contribute to innate host defense by providing bactericidal or bacteriostatic activity prior to subsequent innate and adaptive immune responses [17], [18]. Several antimicrobial proteins and peptides have been detected in the urinary tract from asymptomatic individuals or from those with active UTI; these include cathelicidin, human β-defensin-1 (HBD-1), HBD2, cathelicidin, psoriasin, lactoferrin and RNAse7 [19]–[25]. Defensins are cationic peptides are classified into α-, β-, and θ defensins based upon their sequence homology and cysteine residues [26]. HBD1 is constitutively expressed in epithelia, predominantly in the kidney and female genital tract [21]. HBD2 is induced in response to inflammation or infection and serves to augment epithelial barriers exposed to bacteria or inflammatory mediators [27], [28]. Prior work in bladder epithelial systems has demonstrated that AMP abundance in the epithelial microenvironment is associated with disease severity or inflammation [29]. In humans with UTI, HBD1 and lactoferrin are elevated in urine [30], [31], whereas urinary psoriasin was identified as a biomarker for squamous cell carcinoma of the bladder [32]. A critical role for AMPs in UTI resistance was identified in β-defensin-1-deficient mice, whose bladders were more frequently colonized with *S. aureus* than were the bladders of wild-type mice [33]. In a separate study, urothelial-derived cathelicidin was shown to confer protection against uropathogens, as cathelicidin-deficient mice exhibited a more rapid ascending *E. coli* infection compared to wild-type mice. It was concluded that urinary cathelicidin is derived primarily from the urothelium, as cathelicidin levels did not correlate with urinary leukocyte counts [25]. Collectively, these data demonstrate the importance of AMPs in the urinary tract, and suggest that AMP dysfunction may increase UTI risk.

The interdependence of the urinary microbiota and AMPs in the context of UTI susceptibility has not been investigated. Here, we explored the interrelationship between the urinary microbiota, urinary AMPs and UTI symptom severity in females undergoing POP/UI surgery. We report that the relative abundance and/or frequency of certain members of the urinary microbiota in urines obtained on the day of surgery (DOS) can distinguish between POP/UI subjects who will or will not develop a post-operative UTI. We also report a positive correlation between a greater abundance of urinary HBD1 in subjects with POP symptoms and reduced risk of post-operative UTI. Finally, we investigated potential mechanisms for the variable microbiota between cohorts and determined that AMP activity and hydrophobicity, as well as urinary protease activity, are significantly more robust in individuals whose DOS urine cultures were positive for typical uropathogens or in individuals whose cultures became positive following surgery. Individuals whose DOS cultures were negative and exhibited no signs of UTI following surgery had minimal AMP activity and hydrophobicity, as well as urinary protease activity. Thus, the urinary microbiota and AMP profile have the potential to be used as biomarkers to identify women at increased risk for post-operative UTI prior to POP/UI surgery. Such identification of a high-risk group would facilitate interventional clinical trials aimed at prevention of or improved treatment strategies for UTI.

## Results

### Clinical Assessment and Patient Demographics

Fifty-four women participated in this study by completing an extensive pre-operative questionnaire (**Table 1** **and data not shown**) and providing a catheterized urine specimen (obtained under anesthesia on the day of surgery). Thirteen participants (24%) had positive DOS urine cultures. The rest (n = 41, 76%) had negative DOS urine cultures. Of these 54 women, 10 (18%) developed post-operative urinary symptoms, while 4 (7%) had positive post-operative cultures. Based on DOS and post-operative cultures, we divided the participants into 3 cohorts: those that had positive DOS urine cultures (POS, n = 13, 24%), those that had negative DOS urine cultures but had positive post-operative urine cultures (PostI-UTI, n = 4, 7%) and those that never had a positive urine culture (negative DOS urine cultures and no positive post-operative cultures) (NEG, n = 37, 69%). The culture results from the POS and PostI-UTI cohorts are indicated in **Table 2** and represent the “uropathogen (s)” grown under standard conditions. For this study, we defined a DOS urine culture as a culture with at least 1,000 bacterial colonies forming units per milliliter reported (CFU/ml), and included UTI symptoms (e.g. urinary burning, pain, urgency) and/or microscopic blood in the urine as a positive UTI. The members of each cohort were similar in demographic and clinical characteristics, including race, age, body mass index (BMI), and incidence of diabetes, hypertension, coronary artery disease, and smoking (**S1 Table**). They were also similar with respect to estrogen status, with participants defined as estrogen-positive if they were either pre-menopausal or were taking any source of exogenous hormones, and estrogen-negative if they were post-menopausal and not on hormones. Participants with a positive urine culture at any point in the 6-week peri-operative period (i.e., the DOS and PostI-UTI cohorts) were more likely to experience post-operative UTI symptoms relative to participants with negative urine cultures (NEG cohort) (62% and 50% versus 22%, *p* = 0.024). On the basis of validated symptoms scales, participants in the three cohorts reported similar bother from urinary (UDI) and colorectal (CRADI) symptoms, but differed significantly in prolapse bother (POPDI, *p* = 0.005).

**Table 1:** Comparison of Pelvic Floor Characteristics Based on Culture Status.

	Cohort			
Clinical Factors	Positive DOS culture (POS) N = 13	Negative DOS culture, positive post-operative culture (PostI-UTI) N = 4	Negative DOS culture (NEG) N = 37	*p*-value
Type of Surgery	POP only = 11 (85%) UI only = 0 POP/UI = 2 (15%)	POP only = 1 (25%) UI only = 1 (25%) POP/UI = 2 (50%)	POP only = 16 (43%) UI only = 11 (30%) POP/UI = 10 (27%)	0.06
Pre-operative Urinary Distress (UDI) Score	121	109	109	0.83
Pre-operative Prolapse Distress (POPDI) Score	184	111	108	0.005
Pre-operative Colorectal Anal Distress (CRADI) Score	134	63	92	0.20
% Reported (yes vs. no) Post-operative Urinary Symptoms	8 (62%)	2 (50%)	8 (22%)	0.024

Clinical factors were collected and analyzed between the 3 cohorts by chi-squared analysis or one-way ANOVA, as appropriate. Significance within the type of surgery (POP, UI or POP/UI), pre-operative pelvic floor symptom scores (based on pre-operative questionnaire) or post-operative urinary symptom status is indicated as ***p-*** **value** (α = 0.05).

**Table 2:** Culture results from POS and PostI-UTI cohorts.

Specimen Number	Cohort	Urine Culture Organism	CFU/ml	UTI Symptoms (per patient)	Microscopic blood in urine
690	POS	*E. coli*	>100,000	No	No
868	POS	*S. aureus Corynebacterium spp.*	10,000 5000	No	No
916	POS	*P. aeruginosa*	20,000	No	No
1015	POS	*P. aeruginosa*	50,000	No	No
1018	POS	*E. coli*	50,000	No	No
817	POS	*E. coli*	>100,000	Yes	Yes
1049	POS	*K. oxytoca E. coli*	>100,000>100,000	Yes	Yes
1055	POS	*P. aeruginosa P. aeruginosa* Viridans *Streptococcus* Group B *Streptococcus*	100,000 100,000 25,000 25,000	Yes	Yes
1092	POS	*K. pneumoniae*	1,000	Yes	No
1173	POS	*E. coli*	>100,000	Yes	Yes
1185	POS	*Staphylococcus spp. Actinomyces meyeri Proteus mirabilis*	>100,000>100,000>100,000	Yes	Yes
1201	POS	*Staphylococcus spp. E. coli*	>100,000>100,000	Yes	Yes
1210	POS	*P. aeruginosa* (strain 1) *P. aeruginosa* (strain 2) *Lactobacillus* (strain 1) *Lactobacillus* (strain 2)	>100,000 50,000 6000>100,000	Yes	No
1213	PostI-UTI	*Lactobacillus* spp.	10,000	No	Yes
661	PostI-UTI	*Enterococcus spp.*	1000	No	Yes
835	PostI-UTI	*E. coli* (strain 1) *E. coli* (strain 2)	>100,000 3000	Yes	No
1086	PostI-UTI	Group B *Streptococcus*	12,000	Yes	Yes

Urine culture bacterial counts are represented as colony forming units per milliliter (CFU/ml). UTI symptoms indicated by patient questionnaire (burning, urgency, irritation) or microscopic evaluation for blood. In some cases, two distinct strains of the same species were identified and are indicated as “strain 1” or “strain 2”.

### Distinct Bacterial Taxa Cluster on the Basis of UTI Cohort

**Figure 1:**
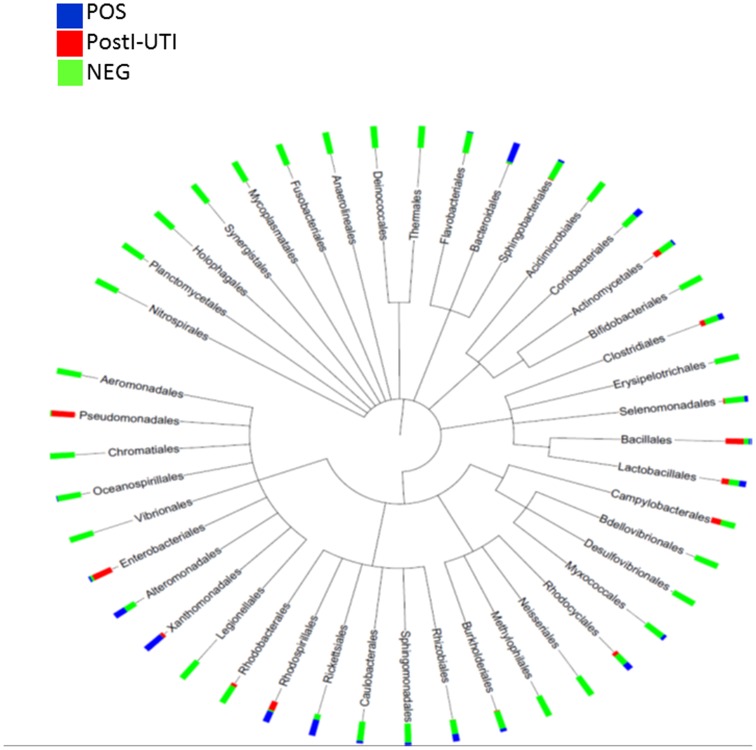
Bacterial diversity correlates with the susceptibility or resistance to UTI. Phylogenetic tree comparing the bacterial diversity at the Order level within the female urinary microbiome. The bacterial diversity was compared between the three cohorts: POS (Blue), PostI-UTI (Red) and NEG (Green).

**Figure 2:**
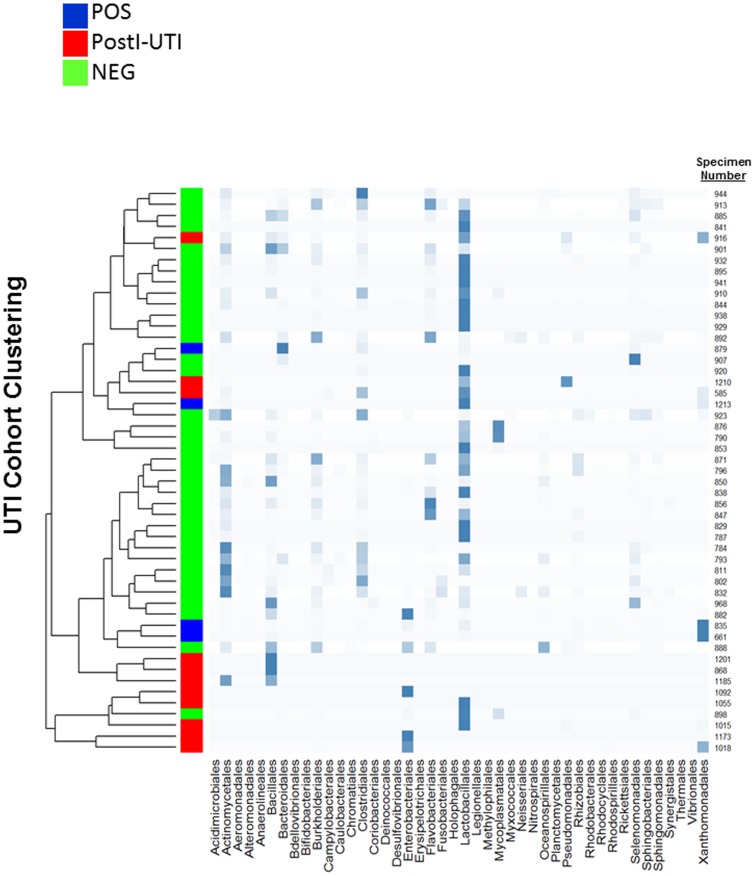
Bacterial abundance and phylogenetic similarity distinguishes the three UTI cohorts. Heatmap representing urine specimens clustered according to their phylogenetic similarities. The abundance of each bacterial Order is represented by the blue boxes, with greater abundance indicated by darker blue squares. Y-axis: Dendogram based on phylogenetic similarities. X-axis: Order. POS (Blue), PostI-UTI (Red) and NEG (Green).

To identify the microbiota in each DOS urine sample, we performed PCR to amplify the V1-V3 hypervariable regions of the 16S rRNA gene and amplicon sequencing on the Roche 454 Titanium platform. From the 48 specimens that did amplify, we obtained 107,532 high-quality bacterial 16S sequences for subsequent analysis: 19,356 from the DOS POS, 5,028 from the PostI-UTI, and 83,148 from the NEG cohorts. We were unable to amplify bacterial 16S sequences from 6 out of 54 specimens. Taxonomy of the high-quality sequence reads was assigned using RDP classifier v2.4 using the default 80% confidence threshold. Taxa in 204 genera, 105 families, 44 orders, 29 classes, and 18 phyla were identified (**Fig. 1**). In all 3 cohorts, some participants had a urinary microbiota that was dominated by a single taxon, representing 80–100% of all assigned reads. For example, POS cohort specimens 868 and 1201 were 99% *Staphylococcus*, a member of the order Bacillales (**Fig. 2** **and data not shown**). POS specimens 1092 and 1173 were 99% *Klebsiella* and *Escherichia*, both members of the order Enterobacteriales. POS specimen 1055 was 99% *Lactobacillus*, while PostI-UTI specimen 1213 was 89% *Lactobacillus*, a member of the order Lactobacillales. This was also true for some NEG specimens. Specimens 787, 841 and 853 were 88–97% *Lactobacillus*. NEG specimen 876 was 100% *Ureaplasma* of the order Mycoplasmatales, while NEG specimen 907 was 100% *Veillonella* of the order Selenomonadales. However, most microbiotas were more complex, containing several genera that comprised at least 5% of the reads [for example, specimens 892 and 916]. Yet, the most complex bacterial community contained only eleven genera that comprised at least 1% of the total reads. Diversity at the family level, as measured by several tests (Shannon, *p* = 0.003; Simpson, *p* = 0.009; Chao1, *p* = 9.1×10^−5^) correlated inversely with both a positive DOS urine culture (POS cohort) and development of a post-operative UTI (which includes all members of the PostI-UTI cohort (4/4) and many members of the POS cohort (6/13)). These relationships also were observed at the order, class and phylum levels (data not shown). In contrast, there were no correlations at any taxonomic level between diversity and any other clinical assessment or patient demographic (**data not shown**).

To assess the relationship between specific bacteria and patient cohort, we analyzed genera abundance. For example, *Lactobacillus* was highly abundant in all 3 cohorts on average, ranging from 16–29% of total 16S rRNA reads in each cohort, but no statistical differences were observed between the cohorts (**Table 3**). In contrast, other abundant genera (>0.5% of total reads in at least one cohort) were found in significantly larger amounts in one cohort relative to another. Several genera were enriched in the PostI-UTI or POS cohorts. Most notably, *Fulvimonas* was highly enriched in the PostI-UTI and POS cohorts relative to the NEG cohort (*p* = 1.13×10^−24^ and 1.23×10^−19^, respectively), while *Dyella* was highly enriched in the PostI-UTI cohort relative to the NEG cohort (*p* = 3.20×10^−10^). *Fulvimonas* and *Dyella* are members of the order Xanthomonadales, which was enriched in the POS and PostI-UTI cohorts (*p* = 5.03×10^−9^ and 8.92×10^−16^, respectively). *Klebsiella* was enriched in the PostI-UTI cohort relative to the NEG and POS cohorts (*p* = 6.65×10^−3^ and 7.79×10^−10^, respectively), while *Escherichia/Shigella* was enriched in the POS cohort relative to the NEG cohort (*p* = 8.99×10^−23^). Both *Klebsiella* and *Escherichia/Shigella* are members of the order Enterobacteriales, which was enriched in the POS cohort (*p* = 2.84×10^−4^). *Pseudomonas* (order Pseudomonadales) also was more abundant in the POS cohort relative to the NEG and PostI-UTI cohorts (*p* = 9.33×10^−19^ and 2.93×10^−4^, respectively), while *Actinobaculum* (order Actinomycetales) was enriched in the POS cohort relative to the NEG cohort (*p* = 9.8×10^−3^).

**Table 3:** Wilcox analysis between Genus and Cohort.

Genus	NEG vs PostI-UTI		NEG vs POS		PostI-UTI vs POS	
	*p* value	Enriched Cohort	*p* value	Enriched Cohort	*p* value	Enriched Cohort
*Aerococcus*			4.35×10^−06^	NEG	4.79×10^−02^	PostI-UTI
*Actinobaculum*			9.80×10^−03^	POS		
*Anaerococcus*	6.86×10^−03^	NEG	1.94×10^−04^	NEG		
*Corynebacterium*	1.35×10^−06^	NEG	2.10×10^−12^	NEG		
*Curvibacter*	4.36×10^−06^	NEG	7.40×10^−07^	NEG		
*Dialister*	2.55×10^−04^	NEG	2.58×10^−03^	NEG		
*Dyella*	3.20×10^−10^	PostI-UTI				
*Escherichia/Shigella*			8.99×10^−23^	POS	1.58×10^−08^	POS
*Facklamia*	7.95×10^−03^	NEG			1.25×10^−07^	POS
*Fastidiosipila*					7.71×10^−05^	POS
*Flavobacterium*	2.46×10^−04^	NEG	1.62×10^−06^	NEG		
*Fulvimonas*	1.13×10^−24^	PostI-UTI	1.23×10^−19^	POS	9.17×10^−03^	PostI-UTI
*Gemella*	8.01×10^−09^	NEG	5.77×10^−13^	NEG		
*Halomonas*	3.88×10^−07^	NEG	8.70×10^−13^	NEG		
*Klebsiella*	6.65×10^−03^	PostI-UTI			7.79×10^−10^	PostI-UTI
*Lactobacillus*	0.115875	PostI-UTI	0.096113	POS	0.614625	POS
*Mobiluncus*	1.10×10^−04^	NEG	1.91×10^−07^	NEG		
*Peptoniphilus*	8.81×10^−03^	NEG	3.92×10^−03^	NEG		
*Propionibacterium*	1.95×10^−04^	NEG	1.64×10^−08^	NEG		
*Pseudomonas*			9.33×10^−19^	POS	2.93×10^−04^	POS
*Streptococcus*	7.52×10^−06^	NEG	4.85×10^−11^	NEG		
*Ureaplasma*	4.82×10^−05^	NEG	2.78×10^−08^	NEG		

For each of the three analyses, all statistically significant correlations are indicated as ***p*** **value**. Non-significant relationships are left blank. The cohort containing a greater abundance of each genus is indicated as **Enriched Cohort**. None of the *Lactobacillus* relationships were significant.

Other relatively abundant genera were enriched in the NEG cohort relative to either the PostI-UTI or POS cohorts. These include members of the orders Actinomycetales (*Corynebacterium, Mobiluncus* and *Propionibacteria*), Clostridiales (*Anaerococcus* and *Peptoniphilus*), and Lactobacilliales (*Aeroccocus, Facklamia* and *Streptococcus*), which also include *Curvibacter* (order Burkholderales), *Dialister* (Selemonadales), *Flavobacterium* (Flavobacteriales), *Gemella* (Bacillales), *Halomonas* (Oceanospirillales), and *Ureaplasma* (Mycoplasmatales). Some genera were enriched in the POS cohort relative to the PostI-UTI cohort (*Escherichia/Shigella, Facklamia, Fastidosipila* and *Pseudomonas*), whereas others were enriched in the PostI-UTI cohort (*Aerococcocus, Fulvimonas*, and *Klebsiella*). *Pseudomonas, Escherichia* and *Klebsiella* were also grown using conventional urine culture (**Table 2**), indicating that we can recover some genera identified from both sequencing and conventional culture-based methods. The tendency for some genera to be associated with one cohort over another suggests the possibility that some members of the urinary microbiota could contribute to lower urinary tract infections, while others could be protective.

### The Distribution of Urinary AMP Levels Differs between Cohorts, while HBD1 Levels in the NEG Cohort Correlates with Pelvic Floor Symptom Severity

**Figure 3:**
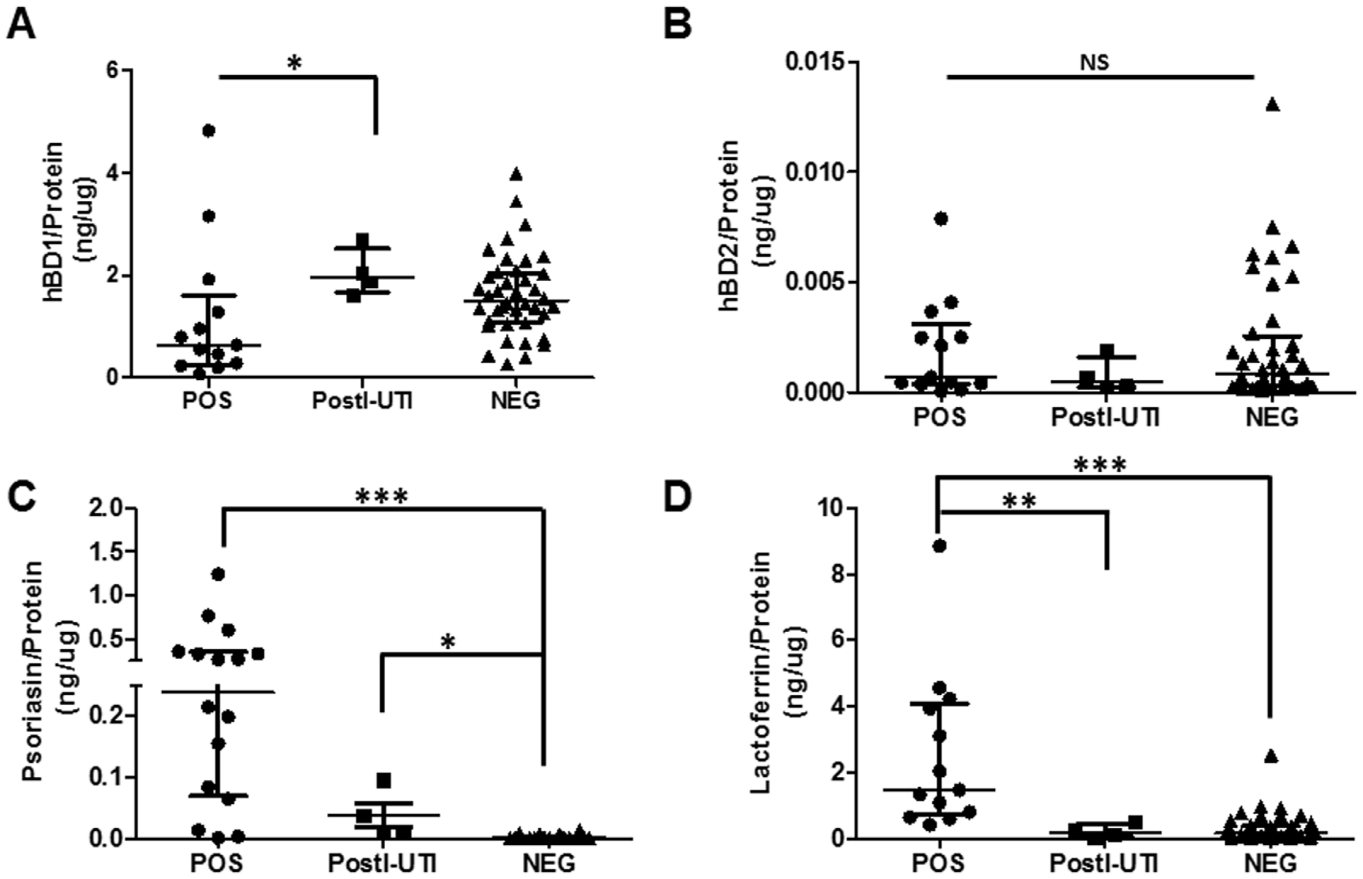
Urinary AMP protein levels in each UTI cohort. Urinary AMP levels were determined by ELISA and normalized to total urinary protein. A. human β-defensin-1 (HBD1) normalized to total protein. B. human β-defensin-2 (HBD2) normalized to total protein. C. Psoriasin normalized to total protein. D. Lactoferrin normalized to total protein. *p<0.05; **p<0.01; ***p<0.001 by One-way ANOVA and Dunn's post-test. Median with interquartile range is shown for each AMP.

One potential reason for UTI susceptibility is differential urinary AMP concentration and/or activity. To determine whether urinary AMPs differed between the cohorts, we used ELISA to measure the protein levels of several candidates (HBD1, HBD2, psoriasin and lactoferrin), which we normalized to total urinary protein (**Fig. 3**). HBD1 levels were significantly lower in the POS cohort relative to the PostI-UTI cohort (*p*<0.05 for POS vs. PostI-UTI) (**Fig. 3A**), whereas HBD2 levels did not significantly vary between the three cohorts (one-way ANOVA and Dunn's Multiple Comparison test, *p*>0.05) (**Fig. 3B**). Psoriasin levels were significantly elevated in both the POS and PostI-UTI cohorts relative to the NEG cohort (*p*<0.001 for POS vs. NEG; *p*<0.01 for PostI-UTI vs. NEG) (**Fig. 3C**), while lactoferrin was elevated in the POS cohort relative to both the PostI-UTI and NEG cohorts (*p*<0.01 for POS vs. PostI-UTI; *p*<0.001 for POS vs. NEG) (**Fig. 3D**). Together, these data demonstrate that POS urine specimens exhibit low HBD1 levels, but high psoriasin and lactoferrrin levels compared to the other cohorts; PostI-UTI urine specimens exhibit high HBD1 and psoriasin levels, but low lactoferrin levels; NEG urine specimens exhibit low psoriasin and lactoferrin levels. This suggests that differences in urinary AMP levels may indicate UTI risk in POP/UI subjects, which may be further influenced by POP/UI status and/or pelvic floor symptoms (**Table 1**).

We next determined if the levels of urinary protein or urinary AMPs might be associated with common pelvic floor symptoms. For this analysis, we computed Spearman's rank correlation between urinary protein or AMP levels and pelvic floor symptom bother. The only significant correlation between symptom severity and urinary protein or AMP levels was identified in the NEG cohort, wherein HBD1 levels positively correlated with prolapse bother (POPDI, Spearman's rank correlation +0.80; *p* = 0.01). We then determined that participants experiencing only POP symptoms (not UI alone nor POP/UI) exhibited significantly lower urinary HBD1 protein levels in the POS cohort, as compared to participants in the NEG cohort (Mann-Whitney U test, *p*<0.05, **data not shown**). Importantly, standard clinical microbiology protocol cultured Gram-negative bacteria from 76% (13 out of 17) of the urines from the POS and PostI-UTI cohorts (**Table 2**). More specifically, 100% (11/11) of POS or PostI-UTI cohort members with only POP symptoms grew Gram-negative bacteria as the single or predominant species. No significant correlations were observed relative to other pelvic floor symptoms, AMPs, or cohort (*p*>0.05). Collectively, these data demonstrate clear associations between urinary HBD1 levels, the susceptibility to post-operative UTI, POP/UI symptoms, and standard urine cultures that grew Gram-negative bacteria.

### AMP Hydrophobicity, AMP Activity and Urinary Protease Activity Cluster by Cohort

**Figure 4:**
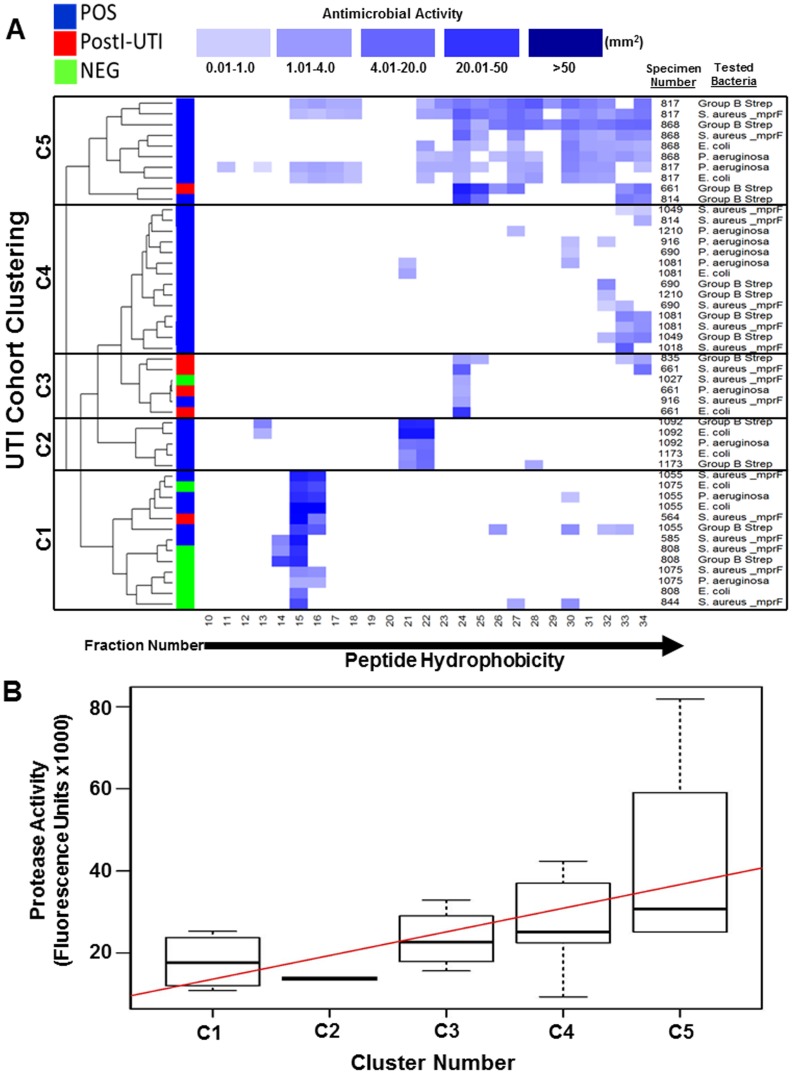
Correlation between urinary AMP activity, hydrophobicity and protease activity. A. Heatmap representing the degree of AMP activity represented by the blue boxes. Proteins and peptides in urine specimens from each cohort were fractionated based upon hydrophobicity. Antimicrobial activity of HPLC-separated urine specimens was measured by a radial diffusion assay. Zones of bacterial growth inhibition were calculated and are represented here in a heatmap such that a darker blue color indicates a larger zone of inhibition. The specimens are clustered based on the Euclidian distances computed from the AMP activities in different HPLC fractions (Cluster 1–5 (C1-C5)). All specimens that exhibited any detectable AMP activity against any of the 4 bacteria tested (*S. aureus*, GBS, *E.* coli or *P. aeruginosa*) are represented here. Fraction number is indicated on the X-axis; hydrophobicity of fractionated peptides increases to the right as fraction number increases (indicated by the solid black arrow). Some specimens generated AMP activity against >1 bacteria, which accounts for the duplication of some specimen numbers on the right Y-axis of the heat map. The color designation is indicated in a key at the top of the heat map. Zone sizes ranged from 0 mm^2^ to 1029 mm^2^. POS urine showed a higher prevalence of antimicrobial activity (12 out of 17 specimens, 71%) than NEG urine (4 out of 11 specimens, 36%) or postI-UTI urine (4 out of 9 specimens, 44%). Specimens are boxed and labeled along the left Y-axis of the heat map (Cluster 1–5). No color indicates undetectable antimicrobial activity within that fraction. POS (Blue), PostI-UTI (Red) and NEG (Green). Specimen number is indicated on the right Y-axis. B. When separated into 5 distinct clusters, antimicrobial activity and AMP hydrophobicity demonstrate a positive correlation with urinary protease activity by a Spearman's rank correlation (R = 0.55, p = 0.010). Protease activity was measured by an Enzcheck protease assay.

Local abundance is only one important factor that determines effectiveness of an AMP. Other factors include peptide charge and degree of hydrophobicity, which dictate an AMP's ability to efficiently kill microbes and stimulate later immune responses [34]. To link AMP hydrophobicity with the degree of AMP activity, we next assessed the functional capacity and hydrophobic characteristics of urinary AMPs. High Pressure Liquid Chromatography (HPLC) was used to partially purify urine specimens prior to analysis of antimicrobial action, which included additional samples collected originally for AMP analyses only. The analyzed specimens included 85% (11/13) of the POS samples, 100% (4/4) of the PostI-UTI and 3% (1/37) of the NEG samples listed in **Table 1**. We also acquired additional specimens for the AMP HPLC analyses, but were limited to AMP analyses only due to limited specimen volume, for a total of 17 POS, 9 PostI-UTI and 17 NEG specimens for the analysis of AMP hydrophobicity and activity. Urinary peptides eluted between 10–55% acetonitrile. Peptides exhibiting greater hydrophobicity eluted from the column into 27 fractions at one-minute intervals with increasing percentages of acetonitrile, which corresponds to fractions 10–35 in **Fig. 4A**. Fractions from each urine specimen were subjected to a radial diffusion assay [35] to assess their capacity to inhibit the growth of typical Gram-positive (*Staphylococcus aureus* or Group B *Streptococcus*) or Gram-negative (*Escherichia coli* and *Pseudomonas aeruginosa*) uropathogens. 2 major categories were apparent: specimens that exhibited no detectable AMP in any fraction (29% (5/17) POS, 56% (5/9) PostI-UTI and 64% (7/11) NEG) and specimens that exhibited various degrees of AMP activity in at least one fraction (71% (12/17) POS, 44% (4/9) PostI-UTI and 36% (4/11) NEG). On the basis of this analysis, 5 distinct clusters became apparent (**Fig. 4A**). Cluster 1 consisted mostly of specimens from the NEG (50%; 3/6) and POS cohorts (33%; 2/6). These specimens exhibited high AMP activity (>20 mm^2^) against at least one tester strain, low peptide hydrophobicity and low diversity, as indicated by the concentration of blue squares primarily in fractions 14–16. Cluster 2 was composed strictly of POS specimens (100%; 2/2), while cluster 3 was a mix of all 3 cohorts (PostI-UTI, 50%, 2/4); POS, 25%, 1/4); and NEG, 25%, 1/4). The specimens in these two clusters tended to have moderate AMP activity (1–20 mm^2^), low diversity and moderate hydrophobicity (fractions 21–22 and 24, respectively). Cluster 4 was composed entirely of specimens from the POS cohort (100%, 6/6). These specimens exhibited low-to-moderate activity (0.01–20 mm^2^), moderate diversity and generally greater hydrophobicity. Finally, Cluster 5 was composed mostly of POS cohort specimens (75%, 3/4). These specimens displayed low-to-moderate activity (0.01–20 mm^2^) and high diversity relative to hydrophobicity.

For clusters 1 and 2, no statistical correlations between AMP activity and hydrophobicity were observed in any of the cohorts. However, statistical correlations between AMP activity and hydrophobicity were identified in several POS and PostI-UTI specimens from clusters 3–5. In cluster 3, significant correlations between AMP activity and hydrophobicity in the PostI-UTI cohort were identified against Gram-positive bacteria (specimen number 835, *p* = 0.04 for GBS), which corresponded to post-operative detection of typical Gram-negative uropathogens (2 distinct *E. coli* strains) by conventional culture methods (**Table 2**). No significant correlations were identified in POS or NEG specimens from cluster 3. In cluster 4, however, significant correlations between AMP hydrophobicity and greater AMP activity against Gram-positive bacteria were identified in several POS specimens (specimen 690 *p* = 0.032 for *S. aureus*, specimen 1049, *p* = 0.003 for GBS, *p* = 0.017 for *S. aureus*; specimen 1081, *p* = 0.018 for both GBS and *S. aureus*), which also corresponded to DOS or post-operative detection of typical Gram-negative uropathogens (e.g., *E. coli* and *K. oxytoca*) by conventional culture methods (**Table 2**). For cluster 5, we identified statistical correlations from every participant, which included 3 from the POS and 1 from the PostI-UTI cohorts. In the POS cohort, we observed statistical correlations between AMP hydrophobicity and greater AMP activity against Gram-positive bacteria (specimen 817, *p* = 5×10^−4^ for GBS, *p* = 1×10^−4^ for *S. aureus*), which corresponded to a DOS culture result of *E. coli* (**Table 2**). We further identified statistical correlations against both Gram-positive and Gram-negative bacteria in one specimen from the POS cohort (specimen 868, *p* = 1.41×10^−8^ for GBS, *p = *2.21×10^−4^ for *S. aureus*, *p = *1.5×10^−4^ for *E. coli*, *p = *2.33×10^−6^ for *P. aeruginosa*), which corresponded to DOS detection of Gram-positive bacteria (e.g. *S. aureus* and *Corynebacterium spp*) (**Table 2**). In the PostI-UTI cohort, we also observed a statistical correlation between AMP hydrophobicity and greater AMP activity against Gram-positive bacteria (specimen 661, *p* = 0.023 for GBS), which corresponded to a post-operative detection of Gram-positive bacteria (e.g. *Enterococcus spp.*) (**Table 2**). Collectively, these data demonstrate that AMP activity and hydrophobicity significantly differs among the NEG, POS and PostI-UTI cohorts. However, statistical correlations between AMP activity and hydrophobicity were only identified in clusters 3–5, which were comprised of POS and PostI-UTI specimens. Furthermore, the frequency of DOS or post-operative culture of Gram-positive vs. Gram-negative bacteria in the POS and PostI-UTI cohorts increased as cluster status increased, suggesting that less AMP activity and more diverse peptide hydrophobicity (e.g. clusters 3–5) in the urine may directly influence UTI risk by Gram-positive or Gram-negative bacteria.

Because proteolysis is a key process in AMP activation, we postulated that an inherent defect in urinary protease activity could result in aberrant processing of AMPs into inactive or more inflammatory peptides. Urine specimens were further clustered based upon their total urinary protease activity to identify correlations between urinary protease activity and AMP activity between the cohorts. Total protease activity tended to be greater in the POS cohort relative to the NEG cohort (**data not shown**). Pattern analysis of the data reveals that cluster 1, comprised of mostly NEG specimens (3/6), is characterized by the lowest degree of protease activity and AMP hydrophobicity, but greater AMP activity. Cluster 2 consists exclusively of POS specimens (2/2), and is characterized by minimal protease activity compared to the other clusters, but greater hydrophobicity relative to cluster 1. Cluster 3 consists mostly of POS and PostI-UTI specimens (3/4), and is characterized by greater protease activity and hydrophobicity relative to clusters 1 and 2, but reduced AMP activity across the fractions. Cluster 4 also consists exclusively of POS urines (6/6), but is characterized by greater protease activity and peptide hydrophobicity, as well as less AMP activity across the fractions relative to clusters 1–3. Cluster 5 consists mostly of POS specimens (3/4), but exhibits the greatest protease activity and diversity of peptide hydrophobicity, which corresponds to less overall AMP activity across all fractions relative to the other clusters. When the clusters from **Fig. 4A** were statistically analyzed to compare the respective protease activity (**Fig. 4B**), the results indicated that cluster 5 exhibited the greatest protease activity (cluster 5: mean 42083.63±27061.81 units) compared to clusters 1–4 (cluster 1: 17815.38±6942.91 units, cluster 2: 13707.50±475.18 units, cluster 3: 23433.88±7391.06 units, cluster 4: 27921.00±11705.70 units). Further statistical analysis identified a significant positive correlation between cluster number and the degree of protease activity by Spearman's rank correlation (R = 0.55, *p* = 0.010). Importantly, this increase in protease activity in cluster 5 parallels the varying degree of peptide hydrophobicity observed in the AMP activity heat map (**Fig. 4A**).

Negative correlations were also observed between urinary protease activity and AMP activity in fractions 15 (Spearman's rank correlation: −0.353, *p* = 0.016) and 16 (Spearman's rank correlation: −0.333, *p* = 0.024), which corresponds with cluster 1 comprised of mostly NEG urine specimens. In contrast, positive correlations were identified in fractions 27 (Spearman's rank correlation: 0.565, *p* = 4.24×10^−5^), 30 (Spearman's rank correlation: 0.482, *p* = 6.8×10^−4^), 31 (Spearman's rank correlation: 0.563, *p* = 4.63×10^−5^) and 32 (Spearman's rank correlation: 526, *p* = 1.8×10^−4^), which consisted of 90% POS specimens. Collectively, these data indicate that the degree of protease activity can dictate the degree of AMP hydrophobicity, and thus activity, in a given urine specimen, which may serve as a mechanism for increased risk for UTI in the POS and PostI-UTI cohorts

## Discussion

Numerous studies of the impact of microbiota on non-urothelial tissues have established dynamic and vital interactions between resident microbiota and host physiology or pathology [10], [12], [19], [36], [37]. Emerging evidence reveals that invading pathogens must compete with the endogenous microbiota, while simultaneously evading host innate immune responses. Given the existence of the newly discovered female urinary microbiota [13], [14], [19], [38], the same is likely true of pathogens implicated in post-operative UTIs. Therefore, pathogenesis of the female lower urinary tract would involve a series of complex and interdependent communications between the host and her urinary microbiota that are not well understood.

The current study links POP symptoms and POP/UI surgery with a positive urine culture on the day of surgery and reveals significant differences in the DOS urinary microbiota of women who developed a post-operative UTI (POS and PostI-UTI cohorts) relative to those who did not develop UTI. For this study, we defined a DOS urine culture as a culture with at least 1,000 bacterial colonies forming units per milliliter reported (CFU/ml), along with UTI symptoms and/or microscopic blood in the urine (**Table 2**). This differs from most other studies in that 10,000 CFU/ml is typically considered a UTI, despite the presence or absence of urinary symptoms (e.g. burning, urgency, pain, blood). False-negative UTI results put patients at-risk for developing secondary complications, such as urinary biofilm formation, pyelonephritis and/or pelvic floor pain and dysfunction. In parallel, using a CFU/ml of 10,000 would not allow for clinicians to distinguish between a true UTI and asymptomatic bacteriuria, which may promote the use of unnecessary antibiotics. The urinary bacteria detected by 16S rRNA sequencing is not due to sample contamination, as we have thoroughly compared the urinary microbiota in clean-catch urine, urine from transurethral catheter, and urine from suprapubic aspirate [7], [13]. Our group has further determined that extended urinary cultures are necessary to grow viable bacteria from urine, as >90% of all bacteria are unculturable by current laboratory techniques. Urine was shown to contain communities of living bacteria that comprise a resident female urine microbiota, which parallel the predominant bacteria detected by 16S rRNA sequencing data from the same subjects [14]. Thus, the bacteria detected in the culture-negative subjects are not due to contamination, but likely represent a “protective” microbiota. Further studies are necessary to determine if intracellular bacterial communities exist in these subjects, or whether the bacteria are planktonic in nature within the urinary tract. The establishment of a urinary microbiome emphasizes the need to redefine “UTI” to include both urinary symptoms and microbiome analyses, in addition to extended culture results [14], as part of the clinical diagnosis of UTI.

Our prior work documented that members of the POS cohort have an increased risk for post-operative UTI [7]. Given that the bladder environment of women with POP is likely different from that of women without POP [7], it is not surprising that the POS cohort was comprised entirely of women who underwent POP surgery. Women with a diverse bladder microbiota appear to be protected from UTI risk both pre-operatively and post-operatively relative to those with a bladder microbiota dominated by a single genus. We propose that POP tends to generate a niche conducive to certain bacteria that either contribute to UTI symptoms or at least act as biomarkers for women at risk for post-operative UTI. These bacteria might be those that were more abundant and/or more frequent in the POS and/or PostI-UTI cohorts. Examples at the genus level include *Escherichia/Shigella*, *Klebsiella*, and *Pseudomonas.* Examples also include *Dyella* and *Fulvimonas*, members of the family Xanthomonadaceae, which includes the emerging Gram-negative urinary pathogen *Stenotrophomonas* [39], [40]. Other examples include *Aerococcus* (Family: Aerococcaceae) *and Actinobaculum* (Family: Actinomycetaceae), which includes the species *A. urinae and A. schaalii*, respectively, two emerging Gram-positive urinary pathogens [41]. Further studies are necessary to identify potential metabolic or environmental factors that allow for bacterial dominance or diversity in POP/UI subjects.

This study also revealed a previously unidentified association between the urinary levels of HBD1 and POP symptoms. Members of the NEG cohort exhibited a positive correlation between HBD1 and POP symptom severity. The relationship between HBD1 and POP symptom severity is intriguing, especially given the linkage between the POS cohort, POP symptoms and surgery, and certain bacterial taxa. These data are important from a clinical perspective, as this indicates that individuals in the NEG cohort who exhibit POP symptoms are likely to have higher urinary levels of HBD1, as compared to individuals with POP symptoms in the POS or PostI-UTI cohort. Furthermore, this suggests that participants in the POS or PostI-UTI cohort experiencing POP symptoms alone may be more likely to develop a Gram-negative UTI, as Gram-negative bacteria were cultured from 92% of subjects with POP alone in these 2 cohorts. Women with significant anatomical distortion due to POP may have altered AMP profiles or characteristics; for example, it may be that HBD1 is protective for women with POP. Therefore, individuals who exhibit POP symptoms and exhibit lower levels of urinary HBD1 would, presumably, be at a greater risk for UTI. Female urine contains two predominant HBD1 bands that are more abundant in pregnant females [21], suggesting that the hormonal milieu (i.e. pregnancy, pre- or post-menopausal) likely influences urinary AMP regulation. We speculate that HBD1 may serve to encourage bacterial tolerance in the urinary tract and discourage the overgrowth of pathogenic or opportunistic microbes under normal conditions. However, subjects in the POS or PostI-UTI cohorts with POP symptoms alone likely exhibit unknown mechanisms that reduce the production of urinary HBD1 and render them more susceptible to post-operative UTI.

Although psoriasin levels were higher in both the POS and PostI-UTI cohorts relative to the NEG cohort, *E. coli* was the most common uropathogen routinely cultured from the urine of participants from the POS and PostI-UTI cohorts. Psoriasin is a calcium-binding protein that normally exhibits potent antimicrobial activity against several strains of *E. coli* by sequestering of Zn^2+^ [32], [42], and has been suggested as a urinary biomarker for bladder squamous cell carcinoma [43]. The observed increase in urinary psoriasin levels in the POS and PostI-UTI cohorts indicates that the activity of urinary psoriasin against *E. coli* may be compromised in these 2 cohorts, which could be due to defects in the urinary ionic environment or bladder squamous cell activity, all of which require further exploration.

The observation that the POS cohort exhibited significantly higher lactoferrin levels compared to the NEG and PostI-UTI cohorts could indicate that some members of the POS cohort may be in the initial stages of a UTI on the DOS, as leukocyte-derived lactoferrin has been found to be a biomarker of UTI [31]. Unlike HBD1, HBD2 and psoriasin, native lactoferrin exerts its antimicrobial action through sequestration of iron [31]. This suggests that elevated lactoferrin in the POS cohort may serve as a compensatory mechanism to block iron acquisition by specific urinary bacteria, particularly *E. coli*, which require iron for survival or virulence and exhibit highly effective scavenging mechanisms for iron acquisition [44]. Alternatively, the elevated levels of urinary lactoferrin in the POS cohort may facilitate the colonization or proliferation of pathogens that rely less on iron availability, or interfere with commensals and beneficial bacteria that require iron for survival. Studies are currently underway to identify potential mechanisms by which the metabolic responses of the urinary microbiome are disrupted in POP/UI subjects.

Although other studies in mice and humans detected cathelicidin in the urine [25], [45], we were unable to detect cathelicidin in urine samples from our participants. We speculate that, due to the nature of our patient population, POP/UI patients might express lower levels of urinary cathelicidin compared to other subsets of UTI-susceptible populations; this speculation warrants further investigation. Furthermore, the ability of AMPs function indirectly as signaling molecules to stimulate chemokine production, modulate dendritic and/or T cell function, promote chemotactic activity and regulate Toll-Like Receptor (TLR) pathways indicates alternative mechanisms by which altered urinary AMP levels may be increasing the risk for PostI-UTI, and is currently being investigated [46]–[48]. Of note, RNAase 7 is currently the most abundant AMP in the urinary tract, specifically in the urothelium of the bladder, ureter, and kidney, and also exhibits potent antimicrobial properties against both Gram-negative and Gram-positive uropathogens [23], [24]. Thus, RNAse 7 expression or activity may also play a key role maintaining a protective urinary microbiota, and should be evaluated in this patient population.

Statistical analyses of urinary AMP and protease activity identified distinct differences between the cohorts that paralleled the degree of urinary peptide hydrophobicity, where greater protease activity and diversity of peptide hydrophobicity was observed exclusively in the POS and PostI-UTI cohorts compared to the NEG cohort. Hydrophobicity and amphipathicity are considered two critical factors for AMPs that target microbial membranes. Further increases in peptide hydrophobicity were found to dramatically reduce overall AMP activity by promoting peptide dimerization in alpha-helical peptides [49]. One mechanism for aberrant peptide hydrophobicity could be an inherent defect in host protease activity, since proteolysis is a key process in AMP activation [50]-[54]. The enhanced urinary proteolyic activity observed in the POS and PostI-UTI cohorts may result in more hydrophobic inactive peptides that permit the colonization and growth of specific bacteria that were found to be more abundant in these cohorts relative to the NEG cohort. Epithelial protease activity is necessary to activate mature AMP proteins and is highly dependent upon the pH [55], yet we did not observe any statistical correlations between urinary pH and protease activity. Host protease production and/or may be enhanced by the urothelium or by infiltrating immune cells in response to the bladder microbiota. Alternatively, bacterial protease activity derived directly from the bladder microbiota could alter host AMP activity, likely as a selective advantage to allow for colonization and/or proliferation of known or emerging uropathogens. For example, proteases derived from a pathogenic strain of *Porphyromonas gingivalis* were found to degrade HBD3 in a dose- and time-dependent fashion *in vitro* [56]. Notably, exposure of HBD3 to culture supernatants derived from *P. gingivalis* diminished HBD3-mediated AMP activity against *S. aureus*, while the presence of specific protease inhibitors increased the antimicrobial efficacy of HBD3 against *P. gingivalis* in identical assays. These data highlight the fact that the antimicrobial capacity of urinary AMPs (host or bacterial) and urinary protease activity directly factors into the susceptibility of POP/UI subjects to UTI.

Together, our data suggest that the urinary microbiota composition (i.e., frequency and/or abundance) and AMP profile (i.e., distribution, activity and/or hydrophobicity) in DOS urine could serve as biomarkers in the future to facilitate the identification of women at risk for post-operative UTI. Although we cannot generalize this finding to other clinical populations, it is unlikely that this population is biologically unique with regard to UTI risk. Although the importance of the urinary microbiota under physiologic or pathologic conditions is largely unexplored, our data now demonstrate an interdependence between specific urinary microbiota and host urinary AMPs with direct implications for UTI susceptibility and POP symptoms in POP/UI subjects. AMPs act both as modulators of microbial colonization and coordinators of host inflammatory processes, and AMPs have been associated with symptom severity in other inflammatory diseases and infection [36], [52], [57]. Thus, the spectrum and abundance of AMPs (host versus bacterial) in the urine likely contributes to both UTI susceptibility and UTI-symptoms (e.g., burning and irritation) by directly influencing the development of microbial communities in the bladder. Although we cannot distinguish between urothelial, immune cell or bacterial-derived AMPs in the urine specimens used in the present study, ongoing work aims to identify the various AMPs present in urine specimens from the three cohorts.

Further insight into the mechanisms by which bladder microbiota and host AMPs communicate during homeostasis and pathologic states will be critical to our understanding of the pathogen-host interactions in the bladder, and facilitate the development of better prevention and/or treatment strategies for UTI in POP/UI subjects and other UTI-susceptible populations. Unfortunately, the clinical criteria for UTI are not well defined. Furthermore, there is no universally accepted, clinically relevant UTI definition that is useful for human research purposes. Commonly, UTI definitions are multi-faceted, requiring symptoms such as urgency, frequency, urinary incontinence, or dysuria *and* a positive urine culture by standard clinical microbiology methods. Yet, patients often experience “UTI symptoms” (i.e., burning, urgency, irritation) despite a negative urine culture, using traditional laboratory procedures and thresholds. This discordance between symptoms and culture status results in diagnostic uncertainty and further contributes to the economic burden associated with UTIs [58], [59].

## Experimental Procedures

### Patient Population and Urine Collection

All Human Participants procedures were reviewed and approved by the Loyola University Chicago Institutional Review Board. We recruited a cohort of participants undergoing POP and/or UI surgery, which was a larger study assessing the presence or absence of the female urinary microbiota [7]. Women were excluded if they were not English speaking, unable to complete the questionnaires, were not undergoing POP/UI surgery, had recurrent UTIs, had recent treatment for UTI or were on active antibiotic treatment for UTI. Urine was collected via trans-urethral catheter prior to administration of one dose of intravenous peri-operative antibiotics. All participants provided written informed consent for research participation. Pelvic floor symptoms can be measured using the validated pelvic floor distress inventory questionnaire (PFDI), which has 3 subscales that detect symptom presence and quantify symptom bother from urinary (urinary distress inventory (UDI)), prolapse (pelvic organ prolapse distress inventory (POPDI)) and colorectal anal symptoms (colorectal anal distress inventory (CRADI)) [60], [61].

### Urine Culture Methods

Routine microbial urine culture consisted of inoculation of sheep blood agar and MacConkey agar plates with 1 microliter of urine and examination of plates for bacterial growth after 24 hours incubation at 35°C in room atmosphere. For this study, we defined a DOS urine culture as a culture with at least 1,000 bacterial colonies forming units per milliliter reported (CFU/ml), along with UTI symptoms and/or microscopic blood in the urine (**Table 2**). To determine the protein detection method most appropriate for protein normalization in urine, we measured several specimens using three different assays: a Bradford assay, a modified Lowry assay, and a bicinchoninic acid (BCA) assay. Protein levels from the three protein assays were compared with protein levels determined by an automated system (Beckman Coulter DXC, Pasadena, CA) in the Loyola University Chicago Health Sciences Campus clinical laboratory. We concluded that the Bradford assay was the most accurate method to measure urinary protein for the studies.

### Pelvic Floor Distress Inventory (PFDI) and UTI Cohorts

All participants completed the long form of the PFDI prior to surgery. During the 6 week post-operative period women were queried on their urinary symptoms at their post-operative visits and a catheterized urine culture specimen was collected from any participants with signs or symptoms consistent with a UTI (i.e. positive urine dipstick, dysuria, frequency, urgency). Post-operative UTI was defined as a urine culture with at least 1,000 bacterial colonies per milliliter. Participants were divided into cohorts based on urine culture status. The three cohorts were those with positive DOS urine cultures, those with negative DOS cultures who developed post-operative UTI, and those with negative urine cultures throughout the 6-week post-operative period.

### Microbial DNA Isolation

Within 4 h of collection, the urine specimens were centrifuged at 5000×g for 10 min and the resulting pellets were re-suspended in DNA stabilization buffer [62]. Swabs were washed with sterile phosphate buffered saline supplemented with DNA stabilization buffer. All specimens were frozen at −80°C until microbial DNA isolation and sequence analysis. All genomic DNA (gDNA) isolations from the urine specimens and from the reagent-only control specimens were performed within a laminar flow hood. Total gDNA was isolated from urines using the Qiagen DNeasy blood and tissue extraction kit (Qiagen) with the Gram-positive bacteria protocol. The gDNA was stored at 4°C until 16S PCR amplification. Amplicon sequencing libraries were quantified by fluorescent Quant-It dsDNA assay (Life Technologies).

### Sequence Processing

The Mothur software (version 1.23.0) [63] was applied to deconvolute the 454 sequence reads into individual samples based on perfect match to the barcode sequences. Primers and barcodes were trimmed from each read and the trimmed sequences shorter than 200 bp were discarded. Low-quality and chimeric sequences were removed with default Mothur parameters. Taxonomic classification (from phylum to genus level) of the sequence reads was performed by the RDP Classifier (version 2.4) [64] with the default 0.8 confidence threshold.

### ELISAs and Protease Assay

AMP concentrations were measured by ELISA; human β-defensin-1 (PeproTech, Cat # 900-K202), human β-defensin-2 (PeproTech, Cat # 900-K172), psoriasin (Circulex, Cat # CY-8073) and lactoferrin (AssayPro, Cat # EL2011-1). Protease activity was assessed with an Enzchek protease assay (Invitrogen, Cat # E6638). All were conducted according to the manufacturers' instructions.

### HPLC purification of urine

Urine was subjected to peptide separation using a Shimadzu HPLC system. Chilled specimens were passed through a C_18_ column (Thermo Scientific) with acetonitrile running buffer to separate urinary peptides based on their degree of hydrophobicity. HPLC was equilibrated in 0.1% trifluoroacetic acid at a flow rate of 2.0 ml/min and fractions were collected at one-minute intervals between 10–55% acetonitrile. Peptide and protein concentration was monitored by UV absorption at 214 and 280 nm, respectively. Respective fractions from three separate runs of each sample were combined, lyophilized, resuspended in 10 µl of sterile ddH_2_0 (Life Technologies), and then vortexed for one hour at 4°C. We first tested recombinant psoriasin and HBD-1 to determine the approximate elution time for known urinary AMPs. As expected, because of their amphipathic nature, both AMPs eluted off of the column at an intermediate concentration of acetonitrile between 40% and 55% acetonitrile (**data not shown**).

### Peptides and Bacteria

Peptides were purchased from Imgenex (San Diego, CA, USA) and 95% purity was confirmed by mass spectrometry. GBS was grown in Todd Hewitt Broth (THB). *Staphylococcus aureus* SA113, *Staphylococcus aureus* Δ*mpr*F [65], and *E. coli* ATCC 25922 were grown in Tryptic Soy Broth (TSB).

### Antimicrobial radial diffusion assay

HPLC fractions were evaluated by radial diffusion assay, as previously described [35], [66], [67]. Antimicrobial activity was analyzed against the microorganisms listed above. Thin plates (1 mm) with 1% SeaKem GTG Agarose (Cambrex Corporation, East Rutherford, NJ) in 0.5% tryptone containing ∼5×10^6^ cells/ml of bacteria were used for the assay. 1 µl water was used as a negative control and applied to separate wells. As a positive control, 1 µl of synthetic CRAMP (64 µM) was applied to separate wells. Plates were incubated overnight at 37°C. The zone of inhibition was quantified by determining the area of bacterial clearance surrounding the center well using Image J Software.

### Statistical analysis

Clinical and demographic factors were collected and analyzed by chi-squared analysis or one-way ANOVA, as appropriate, using SPSS version 19. AMP ELISA data were analyzed using GraphPad Prism, version 5 (GraphPad Software, Inc., San Diego, CA). ELISA data were analyzed by one-way ANOVA with post-tests. *P* values <0.05 were considered significant. Microbial diversity indices were computed from subsampled sequence data, which were performed by subsampling without replacement of 1000 reads from each sample for 1000 times (if a sample has less than 1000 reads in total, all of its reads will be used for analysis without the subsampling step) to avoid bias caused by the different sequencing depths of samples as previously described [68], [69]. Comparison of the bacterial abundance between different cohorts was performed using the *metagenomeSeq* package with its built-in multiple test correction [70]. All the statistical tests were performed with customized scripts implemented with the freely available R software environment (http://www.r-project.org). All of the sequences and associated metadata were deposited to the NCBI Sequence Read Archive under the accession number SRP045483.

## Supporting Information

S1 Table **Participant Demographics.** The members of each cohort were similar in demographic and clinical characteristics, including race, age, body mass index (BMI), and incidence of diabetes, hypertension, coronary artery disease, smoking and estrogen status.
(DOC)
