# Supplementary material for: Interplay between Bladder Microbiota and Urinary Antimicrobial Peptides: Mechanisms for Human Urinary Tract Infection Risk and Symptom Severity
Source: PLoS One. 2014 Dec 8;9(12):e114185. doi: 10.1371/journal.pone.0114185 (PMC4259481; doi:10.1371/journal.pone.0114185)
Supplement: S1 Table — Participant Demographics. The members of each cohort were similar in demographic and clinical characteristics, including race, age, body mass index (BMI), and incidence of diabetes, hypertension, coronary artery disease, smoking and estrogen status. (DOC) [file pone.0114185.s001.doc]

**Table S1. Participant Demographics.**

| **Demographics** | **Positive DOS culture (POS) n=13** | **Negative DOS culture, positive post-operative culture (PostI-UTI) n=4** | **Negative DOS culture (NEG) n=37** | ***p*-value** |
| --- | --- | --- | --- | --- |
| **Age (years)** | 67 (range 44-89) | 60 (range 44-86) | 56 (range 35-84) | 0.08 |
| **BMI (kg/m2)** | 29 (range 23-44) | 26 (range 16-33) | 29 (range 17-48) | 0.69 |
| **Race** | Caucasian = 11 (85%) African-American = 2 (15%) | Caucasian = 4 | Caucasian = 34 (92%) Asian = 1 (3%) Other = 2 (5%) | 0.25 |
| **Diabetes** | 0 | 0 | 2 (5%) | 0.62 |
| **Hypertension** | 4 (31%) | 0 | 9 (24%) | 0.45 |
| **Coronary Artery Disease** | 1 (8%) | 0 | 1 (3%) | 0.66 |
| **Current Smokers** | 0 | 0 | 0 | N/A |
| **Estrogen status** | Positive = 5 (39%) Negative = 8 (62%) | Positive = 2 (50%) Negative = 2 (50%) | Positive = 20 (54%) Negative = 17 (46%) | 0.63 |
